# Schwannome bénin primitif de la plèvre

**DOI:** 10.11604/pamj.2019.33.164.17625

**Published:** 2019-07-03

**Authors:** Rachid Marouf, Ihsan Alloubi

**Affiliations:** 1 Service de Chirurgie Thoracique et Cardio-vasculaire, CHU Mohammed VI, Oujda, Maroc

**Keywords:** Schwannome, pleural, TDM, vidéo-thoracoscopie, Schwannoma, pleura, CT scan, video-assisted thoracoscopic surgery (VATS)

## Abstract

Le schwannome est une tumeur neurogène développée à partir des cellules de Schwann. Dans la région thoracique, le médiastin est le principal site d'apparition du schwannome. Le plus souvent, il s'agit d'une lésion solitaire et la localisation pleurale est extrêmement rare. Nous rapportons un cas de schwannome pleural bénin primitif chez une femme âgée de 44 ans chez qui la lésion a été découverte suite à la réalisation d'un bilan radiologique pour une douleur thoracique et une dyspnée. Le patient a eu résection chirurgicale complète de cette tumeur sous vidéo thoracoscopie. L'étude anatomopathologique a conclu à un schwannome bénin primitif de la plèvre.

## Abstract (en)

Schwannoma is a neurogenic tumor originating from Schwann cells. When considering the thoracic region, it is most commonly found in the mediastinum. It commonly appears as a solitary lesion and pleural involvement is extremely rare. We here report the case of a 44-year old woman with benign primitive schwannoma of the pleura whose lesion was detected after radiological assessment for chest pain and dyspnea. The patient underwent complete surgical resection using video-assisted thoracoscopic surgery (VATS) technique. The anatomopathological study showed benign primitive schwannoma of the pleura.

## Introduction

Les schwannomes (encore appelés neurinomes ou neurilemomes) sont des tumeurs nerveuses bénignes qui se développent à partir de la gaine nerveuse périphérique de la cellule de Schwann, et sont presque toujours solitaires. Ils sont fréquemment retrouvés au niveau du plexus brachial, des gros troncs nerveux des membres, avec une prédilection pour les régions du coude, du poignet ou du genou. Des formes profondes sont rencontrées dans le médiastin postérieur ou dans le rétropéritoine. Il s'agit généralement de lésions bénignes, asymptomatiques, à croissance lente, et surviennent plus fréquemment chez les adultes de sexe masculin [1]. Nous présentons ici un cas de schwannome bénin primitif de la plèvre.

## Patient et observation

**Figure 1:**
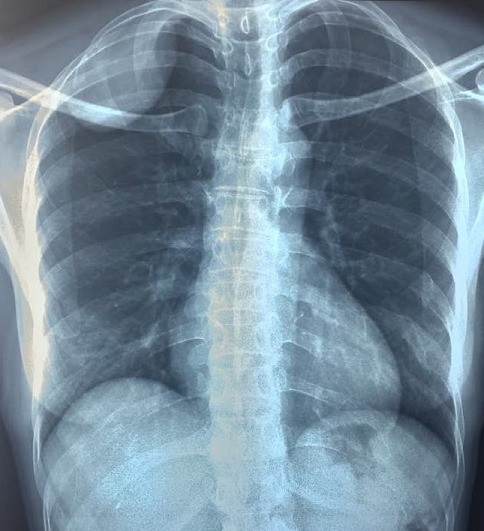
Une radiographie thoracique de face qui montre une opacité à projection apicale droite homogène bien limitée d'allure pariétale thoracique

**Figure 2:**
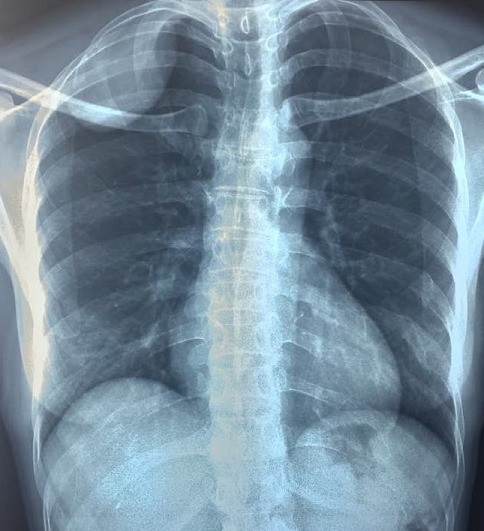
La TDM thoracique objective une masse tissulaire pleurale droite de 5 x 4 x 3,5cm en faveur d'une tumeur fibreuse solitaire ou d'un schwannome ou d'une tumeur maligne

**Figure 3:**
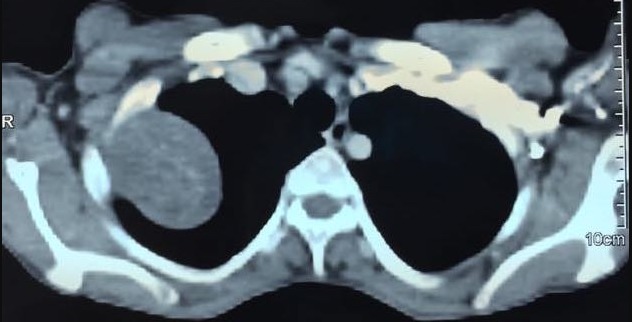
Installation per-opératoire en décubitus latéral gauche, avec mise en place de 2 thoracoport

**Figure 4:**
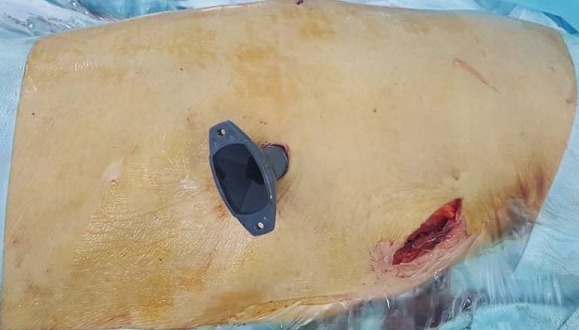
Vue per-opératoire après résection totale de la tumeur sous thoracoscopie

**Figure 5:**
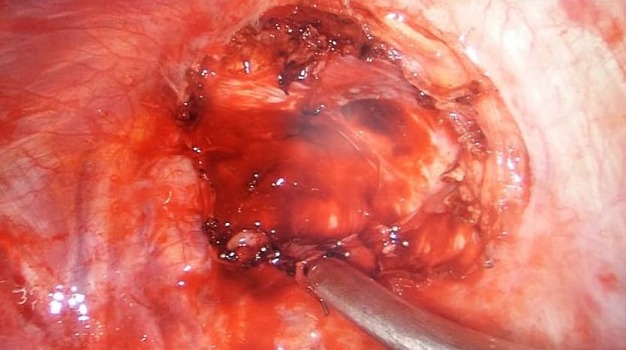
Pièce opératoire

Une patiente âgée de 44 ans, suivie pour problème d'hypothyroïdie depuis 10 ans sous lévothyroxine, opérée pour fibrome utérin il y'a 4 ans, qui présente depuis 1 mois des douleurs latéro-thoraciques droites, une toux sèche et une dyspnée d'effort. Une radiographie thoracique est faite qui a montré une opacité à projection apicale droite homogène bien limitée d'allure pleurale (Figure 1). La TDM thoracique a objectivé une masse tissulaire pariétale thoracique de la plèvre de 5x4x3,5cm de diamètre en faveur d'une tumeur fibreuse solitaire ou d'un schwannome ou d'une tumeur maligne (Figure 2). Après discussion à la réunion de concertation pluridisciplinaire (RCP) il a été décidé de réaliser une résection chirurgicale à but diagnostic et thérapeutique. Sous anesthésie générale avec une intubation trachéale sélective, la patiente a été installée en décubitus latéral gauche. Après mise en place de 2 thoracoports (Figure 3), une thoracoscopie a été réalisée permettant une résection complète de cette masse qui semble naitre de la gaine des fibres nerveuses autonomes de la plèvre et qui respecte le parenchyme pulmonaire (Figure 4). Il s'agissait d'une tumeur encapsulée, ferme, de couleur jaunâtre (Figure 5). L'étude anatomopathologique de la pièce opératoire était en faveur d'une lésion bénigne primitive de la gaine nerveuse périphérique compatible avec un schwannome. Les suites post-opératoires étaient simples sans incident. La patiente a été revue à un an de recul sans aucune récidive.

## Discussion

Les schwannomes pleuraux primaires sont des néoplasmes extrêmement rares [2]. Ils représentent 1% à 2% de toutes les tumeurs thoraciques [3]. Bien qu'il puisse survenir à tout âge, les schwannomes pleuraux affectent généralement les adultes entre la troisième et la sixième décennie. De plus, les hommes sont plus souvent touchés que les femmes [4]. La majorité des schwannomes pleuraux sont bénins, bien que des tumeurs malignes aient été rapportées dans certains cas [5]. Les schwannomes proviennent des cellules de Schwann des gaines des fibres nerveuses autonomes sympathiques ou parasympathiques [6]. Les schwannomes apparaissant à la surface de la plèvre et se développent généralement lentement; et par conséquent la majorité des patients atteints de schwannomes pleuraux sont souvent asymptomatiques et la découverte est fortuite suite à des symptômes non spécifiques. Bien qu'ils soient rarement rencontrés, des cas de schwannomes pleuraux malins ont été rapportés dans la littérature [7]. Ces tumeurs malines sont susceptibles de provoquer un effet de compression sur les structures adjacentes, des symptômes neurologiques et des douleurs thoraciques en raison du caractère agressif de ces lésions et de leur taille plus importante par rapport aux schwannomes bénins [8,9].

Le diagnostic des schwannomes pleuraux est souvent difficile. Un diagnostic définitif ne peut être atteint même avec des modalités d'imagerie avancées et des investigations de laboratoire. Les images radiologiques aident à éveiller les soupçons sur la nature de la lésion et à rétrécir le diagnostic différentiel. Les schwannomes pleuraux doivent être inclus dans le diagnostic différentiel des lésions pleurales solitaires, solides et bien délimitées, qui comprennent notamment les lipomes pleuraux, les métastases pleurales, les mésothéliomes et les tumeurs fibreuses solitaires. Les tests de laboratoire sont généralement dans les limites de la normale. Par conséquent, le diagnostic définitif ne peut être établi que par l'examen histopathologique et la coloration immuno-histochimique du néoplasme. La tomodensitométrie reste la modalité d'imagerie diagnostique pour ces néoplasmes. La tomodensitométrie peut définir la taille, le nombre et l'emplacement exact des lésions. La tomodensitométrie peut également mettre en évidence des composants kystiques et/ou solides de la tumeur. Les schwannomes pleuraux malins présentent des caractéristiques similaires à la tomodensitométrie; Cependant, ils sont généralement associés à la présence de nodules pleuraux, d'épanchements pleuraux et de nodules pulmonaires métastatiques [10]. L'IRM reste plus sensible que la tomodensitométrie pour l'identification du schwannome en dehors des contextes urgents. L'IRM montre une intensité de signal faible à intermédiaire sur les images pondérées en T1. Sur les images pondérées en T2, elle présente une intensité élevée non homogène. Les régions d'intensité très élevée observées sur les images pondérées en T2 de schwannomes correspondent à une dégénérescence kystique avec un tissu fibreux collagène environnant.

Au microscope, les régions Antoni A et Antoni B sont révélées dans la majorité des cas de schwannome pleuraux. Antoni A représente des zones d'hypercellularité avec les corps de Verocay. Les zones Antoni B de l'hypocellularité myxoïde présentent des modifications dégénératives (formation de kystes, hémorragie, calcification, infiltration xanthomateuse et hyalinisation) [11]. Immuno-histochimiquement, les schwannomes pleuraux se colorent généralement de manière diffuse et sont fortement positifs pour la protéine S-100 [11]. Le traitement du schwannome bénin est basé sur l'exérèse chirurgicale compète [12].

## Conclusion

Les schwannomes pleuraux sont des tumeurs extrêmement rares de la cavité thoracique. Les examens d'imagerie et l'étude histopathologique sont nécessaires pour diagnostiquer les schwannomes pleuraux. La prise en charge standard des schwannomes pleuraux consiste principalement en une résection chirurgicale complète par thoracoscopie à chaque fois que cela est techniquement possible avec un suivi continu et régulier.

## Conflits d’intérêts

Les auteurs ne déclarent aucun conflit d'intérêts.
